# Effect of community-based interventions targeting female sex workers along the HIV care cascade in sub-Saharan Africa: a systematic review and meta-analysis

**DOI:** 10.1186/s13643-021-01688-4

**Published:** 2021-05-06

**Authors:** Lydia Atuhaire, Olatunji Adetokunboh, Constance Shumba, Peter S. Nyasulu

**Affiliations:** 1 Division of Epidemiology & Biostatistics, Faculty of Medicine and Health Sciences, Stellenbosch University, Cape Town, South Africa; 2 DSI-NRF Centre of Excellence in Epidemiological Modelling and Analysis (SACEMA), Stellenbosch University, Stellenbosch, South Africa; 3 Cochrane South Africa, South African Medical Research Council, Cape Town, South Africa; 4 School of Nursing and Midwifery, Aga Khan University, Nairobi, Kenya; 5 Department of Population Health, Aga Khan University, Nairobi, Kenya; 6 School of Public Health, Faculty of Medicine and Health Sciences, University of the Witwatersrand, Johannesburg, South Africa

**Keywords:** HIV interventions, Female sex workers, HIV treatment cascade, HIV care continuum, Sub-Saharan Africa

## Abstract

**Background:**

Female sex workers are extremely vulnerable and highly susceptible to being infected with human immunodeficiency virus. As a result, community-based targeted interventions have been recommended as one of the models of care to improve access to HIV services and continued engagement in care. We conducted a systematic review to (1) assess the effect of FSW-targeted community interventions on the improvement of HIV services access along the treatment cascade and (2) describe community-based interventions that positively affect continuation in HIV care across the HIV treatment cascade for FSWs in sub-Saharan Africa.

**Methods:**

We defined the 5 steps that make up the HIV care cascade and categorized them as outcomes, namely, HIV testing and diagnosis, linkage to care, receipt of ART, and achievement of viral suppression. We conducted a systematic search of randomized controlled trials, cohort, and cross-sectional studies done in sub-Saharan African countries and published from 2004 to 2020. The period was selected based on the time span within which ART was scaled up through widespread roll-out of comprehensive HIV programs in sub-Saharan Africa. We reviewed studies with data on the implementation of community interventions for any of the HIV care cascade stage. The data were analyzed using random effects meta-analysis where possible, and for the rest of the studies, data were synthesized using summary statistics.

**Results:**

The significant impact of the community interventions was observed on HIV testing, HIV diagnosis, and ART use. However, for HIV testing and ART use, the improvement was not sustained for the entire period of implementation. There were minimal interventions that had impact on HIV diagnosis, with only one community service delivery model showing significance. Generally, the interventions that had reasonable impact are those that implemented targeted and comprehensive package of HIV services provided at one location, and with unique strategies specific to each cascade stage.

**Conclusions:**

The evidence brought forward from this review shows that the effect of community-based interventions varies across the different stages of HIV care cascade. A broad package of interventions including a combination of behavioral, biomedical, and structural, designed with specific strategies, unique to each cascade stage appears to be more effective, although information on long-term treatment outcomes and the extent to which FSWs remain engaged in care is sparse. There is need to conduct a further research to deepen the assessment of the effectiveness of community-based interventions on HIV care cascade for FSWs. This will enhance identification of evidence-based optimal interventions that will guide effective allocation of scarce resources for strategies that would have a significant impact on HIV service delivery.

**Systematic review registration:**

PROSPERO CRD42020157623

## Background

Female sex workers (FSWs) are 21 times more susceptible to human immunodeficiency virus (HIV) infection than other adults aged 15–49 years [1]. On average, HIV prevalence among FSWs is estimated to be approximately 12% globally [2]. Findings from a systematic review of studies conducted in low- and middle-income countries with medium and high HIV prevalence indicated disproportionately high burden of HIV estimated at 12 to 31% of FSWs living with HIV [3]. In Uganda, HIV prevalence among FSWs is estimated to be 33 to 36% [4] compared to 5.8% in the general population [1] and in Nigeria at 24.5% compared with 4% among adult population aged 15–49 years [5]. HIV infection among FSWs is due to high prevalence of sexually transmitted infections (STIs) and unsafe sex practices with multiple sexual partners attributed to challenging economic circumstances [6, 7]. In addition, punitive environments such as violence, criminalization, stigma and discrimination, social and legal obstacles have been shown to limit access to services for HIV prevention, care, and treatment for FSWs [8, 9]. Various targeted intervention models of care have focused on FSWs with the aim of addressing poor access to HIV services and continued engagement in care [10–13]. In 2016, the World Health Organization (WHO) disseminated consolidated guidelines for key populations including FSWs, with community-based approaches as one of the priority service delivery models [2]. Further, in 2018, the WHO developed a decision framework on differentiated antiretroviral therapy (ART) and, among other strategies, encompassed HIV community responses for FSWs [10]. Among the different types of service provision, community-based service delivery approaches have gained more recognition as an evidence-based intervention to achieve positive health outcomes for FSWs [14, 15]. As such, many HIV/AIDS programs have implemented targeted community service delivery models, and their effectiveness to reducing HIV risk among sex workers has been demonstrated [16, 17].

However, to demonstrate the effectiveness of an HIV intervention depends on the ability to show that a particular intervention can increase access to prevention and treatment services across the treatment cascade [18]. It is therefore critical to measure the performance of community-based interventions across the care and treatment cascade through a series of steps from access to HIV testing and receipt of an HIV-positive diagnosis to successful treatment of their HIV infection. However, such an assessment has not been previously done, despite policy makers’ and donors’ appeal for HIV/AIDS response partners to evaluate the effectiveness of interventions to guide resource allocation and scale up of high impact and sustainable service delivery models [19, 20].

Community-based interventions for FSWs have been assessed in only three previous systematic reviews [14, 17, 21]. The first review aimed to provide evidence on the impact of community empowerment for FSWs on condom use, HIV, and other STI infection. The findings showed that the targeted outcome measure on use of condoms and STI screening was significantly improved due to their association with community-based empowerment approaches [17]. The second review assessed the barriers and facilitators of the implementation of FSW community empowerment programs [14]. The decriminalization of sex work and building of social cohesion among FSWs were identified as facilitators while funding constraints were identified as hindrances to successful implementation. The third systematic review [21] described the nature and structure of targeted community empowerment sexual reproductive health (SRH) interventions and their impact on increased HIV service access for FSW. This review found that, although FSW-dedicated clinics had been established in proximity where FSWs lived and worked and somewhat increased access, very few provided a full package of SRH services for FSWs. All these reviews raised concerns regarding the weak quality of the evidence and recommended further research on the impact of community-based HIV interventions.

Based on the literature review findings, the available systematic reviews have mainly focused on SRH services, and it remains unknown how community-based interventions have been impactful across the HIV treatment cascade. With the extensive recognition that community-based interventions for FSW are an important strategy in HIV response, it is critical to identify and evaluate the uniqueness of specific interventions that may affect FSWs continuation in HIV care. None of the previous studies has systematically presented measurable outcomes attributed to the FSWs-targeted community-based HIV interventions along the HIV treatment cascade. We conducted a systematic review and meta-analysis to find evidence on the effectiveness of community-based interventions that targeted FSWs across the HIV care cascades as outcomes of interest including HIV testing and diagnosis, linkage to care, receipt of ART, and achievement of viral suppression.

### Objectives

1. To assess the effect of FSW-targeted community interventions on the improvement of HIV service access along the treatment cascade including HIV testing and diagnosis, linkage to care, receipt of ART, and achievement of viral suppression.
2. To describe community-based interventions that positively impact continuation in HIV care across the HIV treatment cascade for FSWs in sub-Saharan Africa.

### Research questions

1. Do the FSW-targeted community interventions have an effect on the improved HIV services access across the treatment cascade?
2. Which specific community-based interventions are more impactful to the improved HIV service access across the HIV treatment cascade for FSWs in sub-Saharan Africa?

## Methods and design

The protocol for this systematic review was registered on PROSPERO, CRD42020157623 registration and published [22].

### Definition of FSW community-based service delivery

For the purpose of this systematic review, the terms “community-based service delivery” and “community-based HIV interventions” were used interchangeably. We utilized a working definition suggested by Moore et al. [21] who defined community-based interventions as services that are provided within geographical areas where FSWs live and work. The community-based services considered in this review were those that had been implemented in various ways including (i) those that provided supportive services to the public health facilities such as linking the clients to the health system for HIV care, (ii) routine outreach services, (iii) targeted FSW clinics based in hotspots, and (iv) stand-alone community services without a link to the formal public or private health facilities.

### Definition of female sex work

In this review, FSWs were defined as women who self-identify as sex workers and exchange sex for money, engage in transactional sex, or exchange sex for other gifts and commodities.

#### Eligibility criteria for considering studies for review

We included randomized control trials (RCT), cross-sectional surveys, and cohort interventions conducted in sub-Saharan African countries from 2004 to 2020. We also reviewed unpublished quantitative data from reports and policy documents published within the region and period. The period was selected based on the time span within which ART was scaled up through widespread roll-out of comprehensive HIV programs in sub-Saharan Africa [20].

### Study participants and intervention

To be included in the review, a study had to have evaluated a community-based HIV intervention for FSWs regardless of age and reported its effectiveness on one or more cascade stages. Studies that reported on community-based HIV services provided for FSWs in addition to other groups were also included if data was disaggregated and reported by sex work status.

### Outcomes

In order to be eligible for inclusion in the review, studies should have measured and reported the performance of the community-based HIV interventions at baseline/end-line or multi-arm design. Studies that had set performance targets for one or more treatment cascade stage and reported on outcomes post-intervention were also eligible. In this review, the HIV care cascade stages included HIV testing and diagnosis, linkage to care, receipt of ART, and achievement of viral suppression. Although retention in care is one of the cascade stages, it was intentionally not assessed in this review because of a lack of consensus about how to best measure retention or continuity in HIV [23, 24]. The outcomes were defined following one of the two ways CDC monitors the continuum of care [25]. We followed diagnosis-based HIV care continuum definition that shows each step as a percentage of the number of people living with diagnosed HIV.

HIV testing was defined as reported change (increase or decrease) in the number of FSWs that accessed HIV testing in a community-based HIV testing program; HIV diagnosis was defined as the proportion of FSWs diagnosed with HIV among those tested; linked to care was defined as the proportion of participants who got diagnosed versus the proportion of those that accessed HIV care services, categorized as the completion of a first medical clinic visit after HIV diagnosis; receipt of ART was defined as the proportion of participants who initiated ART, among those who were diagnosed with HIV; and achievement of viral suppression was defined as the proportion of participants who attained viral suppression among those diagnosed with HIV.

### Search strategy

We used a search strategy for electronic bibliographic databases, bibliographies of included articles, and grey literature sources. We developed a comprehensive set of search terms subjectively and iteratively, checking Medline (PubMed) through September 2020 to identify controlled vocabulary (MeSH) terms related to our topic, and identified keywords based on our knowledge of the field. Medline search terms were adapted for other electronic databases to conform to their search functions. The following electronic databases were searched using the date range 2004 to present: Medline (PubMed), CINAHL (EBSCO Host), Science Citation Index Expanded (SCI-Expanded) and Social Sciences Citation Index (SSCI) both from the Web of Science, Scopus (Elsevier), and Cochrane Library, including the Cochrane Central Register of Controlled Trials (CENTRAL). Websites of Joint United Nations Programme on HIV/AIDS (UNAIDS) and WHO were searched for additional reports of sex work programs. In addition to the electronic search, we searched for grey literature to identify any relevant unpublished reports. We also checked the reference lists of relevant articles for additional citations and used the “related citations” search key in PubMed where we identified similar papers. Search results were managed using specialized bibliographic software (Endnote).

### Search terms

The following terms were entered into all data bases:

HIV Infections[MeSH] OR HIV OR “hiv infect*” OR “human immunodeficiency virus”) AND (“HIV treatment cascade” OR “HIV continuum of care”) AND (“Community Health Services”[Mesh]) OR “Delivery of Health Care”[Mesh] OR “Health extension worker*” or “Community led” OR “Community Implementer” OR “community worker*” OR “lay health worker*”) AND (“Sex Workers”[Mesh] OR “Sex Work”[Mesh] OR prostitut* OR “exchanging sex” OR “sex trade ”) AND (“Africa South of the Sahara”[Mesh] OR sub-saharan africa*)

### Selection of eligible studies

Titles and abstracts were screened by two reviewers independently (LA, OA) and harmonized the differences by consensus on the studies eligible for full text screening. All full text articles were assessed for relevance by the same two reviewers independently (LA, OA), and determined final studies that were eligible for inclusion for the systematic review. Disagreements were resolved by mutual consensus and by consultation with the third reviewer (PN).

### Data extraction and management

Data were extracted using a standardized tool developed based on the Cochrane format data collection form for intervention reviews [26]. The developed tool was piloted by two reviewers (LA, OA) independently on a random sample of two articles, and the tool was revised accordingly. For all eligible studies, the same authors extracted data and jointly reviewed the extracted information for harmonisation. Discrepancies in the extracted data were resolved through discussion, consensus, and involvement of a third reviewer (PN) when necessary. The following data was collected from each included study:

We collected data on study identification which included title, author, and year of publication; and characteristics of studies such as study design, target group, description of interventions, type of comparison, type of outcome measures, unit of allocation (individuals or clusters), period of study, duration of follow-up, setting including country of study (locality and social context), method of participant recruitment, random or non-random allocation of participants, baseline imbalances, sample size, and mean age. Other areas for which we collected data on included interventions such as specific type of community-based service delivery interventions per arm where it was applicable and co-interventions. We collected data on outcome measures including number or proportions of FSW outcomes (HIV testing and HIV diagnosis, linked to care, receipt of ART and achievement of viral suppression) at baseline and end-line and time points measured. There was also additional information collected on facilitators and challenges of implementing community-based interventions and limitations of the studies.

### Assessment of risk of bias

Risk of bias for randomized controlled trials was assessed using an adapted Cochrane Collaboration’s tool for assessing risk of bias. We used the tool to assess sequence generation, allocation concealment, blinding of participants and personnel, blinding of outcome assessment, incomplete outcome data, selective outcome reporting, and other sources of bias [26]. For cross-sectional and cohort studies, risk of bias was assessed, and study quality rated using the adapted Newcastle Ottawa scale (NOS) [27]. The Ottawa scale included the following items: (i) representativeness of study sample and ascertainment of the exposure, (ii) comparability of cohorts on the basis of the design or analysis controlled for confounders, (iii) outcome assessment and adequacy of follow-up period. Publication bias was assessed by visually inspecting funnel plot asymmetry and by including study size in the logistic model.

### Data analysis

The data were analyzed using random effects meta-analysis. The main characteristics of included studies were synthesized using summary statistics to describe characteristics such as mean (standard deviation) and frequencies. For each step of the cascade together with the extracted community HIV service delivery interventions, the proportions with exact binomial 95% confidence intervals (CI) were calculated and presented in forest plots.

The homogeneity of the results was calculated by means of the Chi-square test, and the *I*^*2*^ was used to describe the percentage variation across included studies. We planned to explore substantial heterogeneity (*I*^2^>50%) by subgroup analysis; however, the findings did not warrant this. Meta-analyses with substantial heterogeneous results involve few studies and do not warrant subgroup analysis. All analyses were done in STATA version 16.0 (StataCorp. Stata Statistical Software: release 16. College Station, TX).

## Results

### Summary of key findings

Overall, this review found evidence on the impact of selected community-based intervention packages on FSWs continuation in HIV care across the HIV care cascade. The significant impact of the interventions was observed on three cascade stages namely: HIV testing, HIV diagnosis, and ART use. However, for HIV testing and ART use, the improvement was short-lived in that the retention on ART and improved access to HIV testing was not sustained for the entire period of implementation. There were limited impactful interventions for HIV diagnosis with only one community service delivery model showing significance. Generally, impactful interventions were those that implemented targeted and comprehensive package of HIV services provided at one location within places where FSWs worked and lived, and with unique service delivery models for specific cascade stages. This review also found that community-based interventions led to the improvement of linkage to care and viral load suppression to undetectable levels; however, the improvement was not significant. In addition, the results showed that many of the projects were small-scale, research-based, and have limited time-bound implementation periods. A few non-research based and large-scale HIV prevention efforts had limited systematic means of monitoring outcomes along the HIV care cascade and therefore, less methodical attention to constantly reviewing the effectiveness of interventions and altering delivery strategies accordingly.

### Characteristics of included studies

**Fig. 1:**
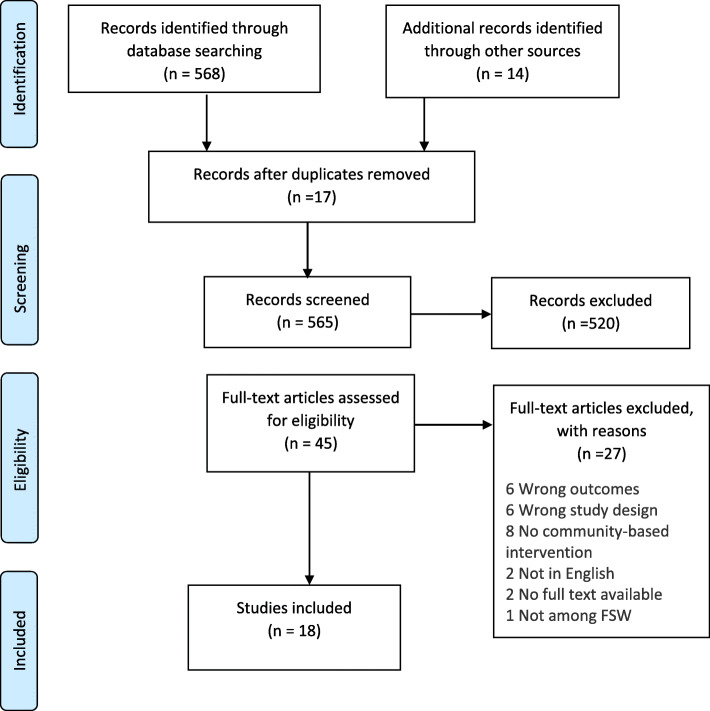
PRISMA flow diagram of selection process

Our literature search yielded 582 articles, and after removing duplicates, we remained with 565 studies. Following screening of the titles and abstracts, we retained 45 studies for full text review. After thoroughly reading the remaining 45 articles, 27 studies were excluded for the following reasons: six articles had reported outcomes that did not meet our eligibility criteria, six did not meet the study design criteria, eight studies had not reported community based interventions, two articles were not written in English, two had either not targeted FSWs or not reported disaggregated data for FSWs, two articles were abstracts from conferences and we failed to access the required information, and one had not been conducted in Sub-Saharan Africa (Fig. 1).

The remaining 18 studies were found eligible for inclusion in the review (Table 1). The study designs were randomized controlled trials (*n* = 3), cross-sectional studies (*n* = 10), and cohort studies (*n* = 4) and quasi-experimental study (*n* = 1). These studies were conducted in seven sub-Saharan African countries (Table 1). Most of the included studies were from Zimbabwe (*n* = 5) [30–32, 40, 41], Tanzania (*n* = 3) [35, 44, 45], and Kenya (*n* = 2) [34, 39], and 5 studies of which each was from Guinea (*n* = 1) [28], Zambia (*n* = 1) [29], Burkina Faso (*n* = 1) [33], Uganda (*n* = 1) [42], and South Africa (*n* = 1) [43].

**Table 1:** Characteristics of selected studies

(#Ref) Author (year)	Country	Study aim	Study design	Sample size and participant selection
[28] Aho et al. (2012)	Guinea	To describe the acceptability and consequences of VCT among a stigmatized and vulnerable group, female sex workers (FSWs), in Conakry, Guinea	Cross-sectional study	Randomly selected 421 at FSW at baseline and 223 at end line. Recruited through private or public centers providing adapted healthcare services (AHS) for FSWs
[29] Chanda et al. (2017)	Zambia	To evaluate the effect of 2 different health system mechanisms (the active approach of peer-based HIV self-test) for HIV self-test delivery compared to referral to standard HIV testing	A 3-arm 1:1:1 cluster randomized trial	Total randomized per arm (320,316 and 329). Peer educator recruitment of social network via direct contact and referral
[30] Cowan et al. (2019)	Zimbabwe	(i) To present the current impact that engagement in the Sisters program has on HIV incidence, prevalence, and control in FSW. (ii) To describe the patterns and characteristics of sex work among FSW in Zimbabwe (iii) To assess the potential for wider population impact of sex worker program by modelling the impact on HIV incidence of eliminating transmission through FSW	Cross-sectional study	Use of program data of 5083 FSW recruited through respondent-driven sampling surveys through three studies conducted in 19 sites: in 2011 to 2015; 2013 to 2016 and 2017
[31] Cowan et al. (2017)	Zimbabwe	To describe the HIV diagnosis and care cascade among FSW in Zimbabwe.	Cross-sectional study	Respondent-driven sampling surveys of FSW in 14 sites. Recruited 2722 women, approximately 200 per site as the baseline for a cluster-randomized controlled trial investigating a combination HIV prevention and care package.
[32] Cowan et al. (2018)	Zimbabwe	To assess the efficacy of a targeted combination intervention for female sex workers in Zimbabwe.	Cluster-randomized trial from 2014 to 2016	Randomly assigned 14 clusters (1:1) to receive usual care cluster (*n* = 3612) and an intervention cluster (*n* = 4619)
[33] Huet et al. (2011)	Burkina Faso	To describe the long-term virological, immunological, and mortality outcomes of providing highly active antiretroviral therapy (HAART) with strong adherence support to HIV-infected female sex workers (FSWs) in Burkina Faso and contrast outcomes with those obtained in a cohort of regular HIV-infected women.	A prospective observational study nested within the Yerelon open cohort of high-risk women	47 FSWs and 48 non-FSWs recruited through a network of peer educators and followed up at a dedicated clinic located within a public health facility.
[34] Kelvin et al. (2019)	Kenya	To assess whether informing female sex workers about the availability of HIV self-testing at clinics in Kenya using text messages would increase HIV testing rates	Cohort study	A sample of 2196 female sex workers selected from electronic records.
[35] Kerrigan et al. (2019)	Tanzania	To determine the impact of a community empowerment model of combination HIV prevention (Project Shikamana) among female sex workers (FSW) in Iringa, Tanzania.	A prospective community-randomized trial conducted in 2 communities matched on population size	Identified all active sex work venues (164 in total) in the 2 study communities and enrolled 496 FSW through a time-location sampling
[36] Lafort et al. (2018)	South Africa, Mozambique, and Kenya	To enhance uptake of SRH services by FSWs through an implementation study	Cross-sectional study (in the context of an implementation research project)	400 FSWs recruited by respondent-driven sampling
[37] Lillie et al. (2019)	Burundi, Cote d’Ivoire, and DRC	To identify KP that had a new HIV diagnosis so that they could be linked to life-saving treatment for epidemic control	Quasi-experimental study	929 FSWs sampled. Selection was done through distribution of coupons by peer
[38] Lafort et al. (2016)	South Africa, Mozambique, and Kenya	To identify gaps in the use of HIV prevention and care services and commodities for female sex workers with the aim of improving SRH services.	Cross-sectional survey (in the context of an implementation research project)	Used RDS to recruit 400 sex worker in Durban, 308 in Tete, 400 in Mombasa, and 458 in Mysor
[39] Luchters et al. (2008)	Kenya	To evaluate the impact of 5 years of peer-mediated STI/HIV prevention interventions among FSW in Mombasa, Kenya	Pre- and post- intervention cross-sectional surveys	Initial respondents (seeds) were identified from FSW work places, with subsequent participants recruited using snowball sampling.
[40] Napierala et al. (2018)	Zimbabwe	(1) To compare engagement in services and the HIV care cascade among FSWs aged 18–24 years compared with those aged 25 years and older. (2) To explore factors associated with young FSWs’ engagement in HIV services.	Cross-sectional survey	Sampled 2722 FSW through respondent-driven sampling from 14 communities
[41] Ndori-mharadze et al. (2018)	Zimbabwe	To compare key indicators related to FSW health-seeking behavior in 2011 and 2015 in three sites and explore whether observed differences might be linked to the delivery of intensified community mobilization.	Cross-sectional study	870 FSW sampled in 2011 and 915 in 2015. FSWs were selected as seeds of the 2015 RDS survey, and also reviewed program data from the Sisters’ clinics between 2010 and 2015.
[42] Pande et al. (2019)	Uganda	To assess preference and uptake of the current community-based HIV testing service delivery models that are used to reach FSW and identify challenges faced during the implementation of the models.	Cross-sectional study design	Used cluster sampling for hotspot selection and recruited 72 FSWs in each cluster
[43] Schwartz et al. (2017)	South Africa	To assess engagement in the HIV care cascade and correlates of ART use among a sample of South African FSWs.	Cross-sectional study	Selection was done through RDS by selecting seeds to represent FSWs across ages, race, and locations
[44] Tun et al. (2019)	Tanzania	To examine differences in treatment outcomes between the intervention and comparison arms.	Quasi-experimental prospective cohort study	309 (intervention) and 308 (comparison) sampled at baseline. FSW selected randomly through community-based HTC in hotspots, directly contacting former Sauti FSWs and use of brochures
[45] Vu et al. (2020)	Tanzania	To increase linkage to and retention in antiretroviral therapy (ART) care, by piloting a community based, ART service delivery intervention for female sex workers	Quasi-experimental prospective cohort study	309 (intervention) and 308 (comparison) followed from baseline. FSW selected randomly through; community-based HTC in hotspots, directly contacting former Sauti FSWs and use of brochures

There were two multi-country studies conducted in Burundi, Cote d’Ivoire, and DRC (*n* = 1) [37], and South Africa, Mozambique, and Kenya (*n* = 1) [36]. All studies in the review were conducted among FSWs, with two studies that included non FSWs and MSM. The first study compared uptake of HIV services among FSW and non-FSW [33], and the second one also included MSM [37] but reported disaggregated results for FSW.

The common implementation models of community interventions included (i) promotion of FSW participation with client-led approach, (ii) intense community mobilization approaches through direct service delivery to places where FSW live and work, and (iii) application of technological innovation (i.e., text messaging) as a tool for mobilization of increased uptake of HIV services. Other interventions also focused on provision of comprehensive package of services using a one-stop shop approach in the community, peer-to-peer implementation approaches, and provision of static services in clinics based in hotspots of FSWs.

The vast majority of the studies focused on traditional FSW service delivery approaches that included peer-focused approach of mobilization, condom promotion, HIV testing, and promotion of regular STI screening (Table 2). Only six studies measured at least two or more HIV cascade outcomes downstream of HIV testing to viral suppression among FSWs at the end of the implementation period of the community-based interventions [28–30, 35, 36, 41].

**Table 2:** A summary table showing impact of community HIV intervention on continuation in HIV care across the treatment cascade, extracted from the included studies

Cascade step	Combined interventions that showed significant impact	Evidence
HIV testing	• Partnership with KP NGOs/CBOs based in the community/hotspots to deliver HTS services on behalf of national programs • Peer educator direct distribution of HIV self-test kits • Repeated use of text messaging and communication on what’s up by peers informing FSW about the availability of testing services in the community • Adapted health care: creation of FSW safe spaces and integration of targeted FSW HIV services in the general health care (e.g., STI screening and treatment, lubricants and condoms, direct escort by FSW peers within a public facility) • Provision of testing through night clinics (bars, brothels, DICs) • Full time provision of testing at clinics based in hotspots • Strengthening support networks FSW CSOs to encourage health-promoting behavior • Venue-based peer education, free condom distribution, and HIV counseling and testing;	Chanda et al. [29] Kelvin et al. [34] Aho et al. [28] Lafort et al. [38] Pande et al. [42]
HIV diagnosis	Enhanced peer outreach approach: • Use of paid outreach peers that have not worked as peers before to find new FSWs from their network • Use of short-term incentivized peer support to reach their hard to reach contacts-FSWs	Lillie et al. [37]
Linkage to care	None	
ART use:	• NGO-initiated FSW-targeted mobile clinical services • Provision of services at a community-led drop-in center • Training of health workers in FSW-friendly approaches • Provision of HIV services in the community clinic by a professional health provider • Extending operating days at community based clinics with flex working hours • Provision of broad package of HIV service offered in clinics based in hotspots • Provision of on call services where FSW can consult anytime • Police sensitivity trainings, violence prevention, and campaigns for anti-stigma and discrimination	Kerrigan et al. [35] Cowan et al. [32] Napierala et al. [40] Pande et al. [42]
Viral suppression	None	
**Interventions that showed a positive effect but with non-significant impact**		
Linkage to	• Enhancing referral mechanisms to the neighboring public health facilities by paying stipend for peers. • Financial facilitation of FSW focal persons based at public health facilities • Establishment and incentivized peer referrals to the DICs • Creation of a safe space at a public health facility in a community without a FSW DIC • Conducting sensitivity trainings to all service providers including the non-professional staff within the clinics • Peer referrals and linkages at the clinics based in hotspots • Behavior change communication to educate and improve health-seeking behaviors • Extended hours of work to evenings, night, and weekends • Mobile HIV services to mitigate transport issues	Chanda et al. [29] Kerrigan et al. [35] Pande et al. [42] Lafort et al. [36]
Viral suppression:	• Usual HIV services augmented with additional community mobilization activities aimed at raising awareness of the benefits of ART. • Building leadership skills among FSW groups • Participation of FSW groups in selecting their fellow FSW adherence supporters • Adherence training sessions for the FSW adherence supporters • Mobile telephone messaging reminders for ART adherence • SMS and follow-up phone to support clinic attendance. • Empowering FSW to improve retention in care by targeting improved individual client-oriented practices	Cowan et al. [32] Kerrigan et al. [35] Napierala et al. [40]

### Risk of bias in included studies

#### The risk of bias in the included RCTs

Selection bias of allocation sequence generation was low in two studies and unclear in the remaining two. Allocation of concealment was unclear in three studies and low in one. In two RCTs, blinding of participants and personnel and blinding of outcome assessment was low and unclear in the remaining two studies. The risk of reporting incomplete data due to attrition and selective outcome reporting was low in three studies, high in one study, and unclear in one study. The risk of bias in all RCTs was highest due to other forms of bias such that all had high risk due to reliance on self-report for outcomes and short periods of intervention implementation with uncertain population effect. Other risk of bias were also related to low sample sizes including testing the intervention on limited number of FSW and FSW communities.

#### The risk of bias assessment in cohort studies

One cohort study had low risk of bias in relation to selection of representative samples and justification for case and control selection. However, for two cohort studies, the risk of bias was unclear as information on justification for selection of control and cases was not provided. Never the less, both studies demonstrated that participants were not exposed to the intervention before the start of the study. All cohort studies had adequate measures of outcome assessment by using validated measurement scales, measurement of ART use by pill count rather than self-report, and had relatively long follow-up periods of 6 to 12 months.

#### The risk of bias for cross-sectional studies

All the nine included cross-sectional studies had low risk of bias in regard to selection of sample size and its representativeness. However, the overall score between studies varied, but the scoring grades weighed within the acceptable range of 4 to 6. In regard to risk of bias of assessing whether confounding factors were controlled, this information was not indicated in all the studies apart from one cross-sectional study which reported that post hoc pairwise comparison tests were conducted with RDS-adjusted weights while adjusting for the confounding effect. Lastly, one study did not describe outcome assessment; therefore, its risk of bias was unclear. However, in the rest of eight cross-sectional studies, risk of bias for outcome assessment was low as there was adequate description of the validated measures used to control for risk of bias. These included reviews of medical records and health assessment by qualified staff among others. All studies used statistical tests to control for bias in individual studies.

### HIV testing services

HIV testing was done in two randomized control studies conducted in Kenya [34] and Zambia [29]. The participants in the intervention arms for both studies were more likely to test for HIV than the standard of care arms. At 1 month’s follow-up, Chanda et al. [29] registered 94.9% of HIV testing; however, the testing rates dropped to 84.1% at 4 months of follow-up in the intervention arm. Similarly, in an RCT [34], participants in the intervention arm were significantly (OR 1.9, *p* = 0.001) more likely to test for HIV, (81, 10.8%) compared to those in the enhanced standard of care (46, 6.1%) and usual standard of care (43, 6.2%) (OR 1.0 (*p* = 0.972)).

Three cross-sectional studies also reported HIV testing as an outcome [28, 36, 42]. In a study conducted in Guinea [28] where adapted HIV care services were provided to FSW, there was 100% acceptance to HIV testing although there were reports of coercion by managers of FSW worksites affecting voluntary consent for an HIV test. Equally, Lafort et al. [36] conducted context-specific targeted community intervention in three cities: Durban in South Africa, Tete in Mozambique, and Mombasa in Kenya. In all the three cities, among all services provided, the greatest effect was on uptake of HIV testing, increasing from 40.9 to 83.2% in Durban, 56.0 to 76.6% in Tete, and 70.9 to 87.6% in Mombasa. Finally, a cross-sectional study [42] conducted in Uganda compared three models of community interventions and assessed model preference determined by increased access and utilization of HIV testing services. This study showed that static clinics based in FSW hotspots were preferred (72% (279/390)) compared to 25% (98/390) that used outreaches and 3.3% (13/390) that used peer-to-peer mechanisms to have an HIV test. These models were implemented and assessed over a period of 12 months.

### HIV diagnosis

**Fig. 2:**
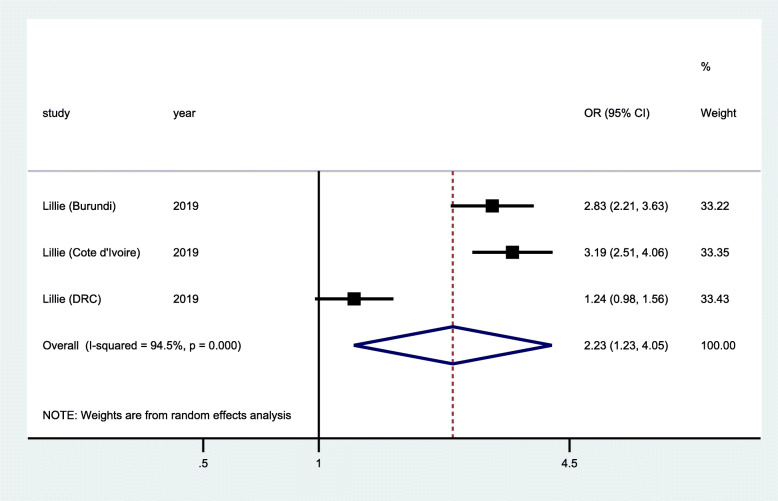
Forest plots of quasi-experimental study with data on community-based interventions for HIV diagnosis

**Fig. 3:**
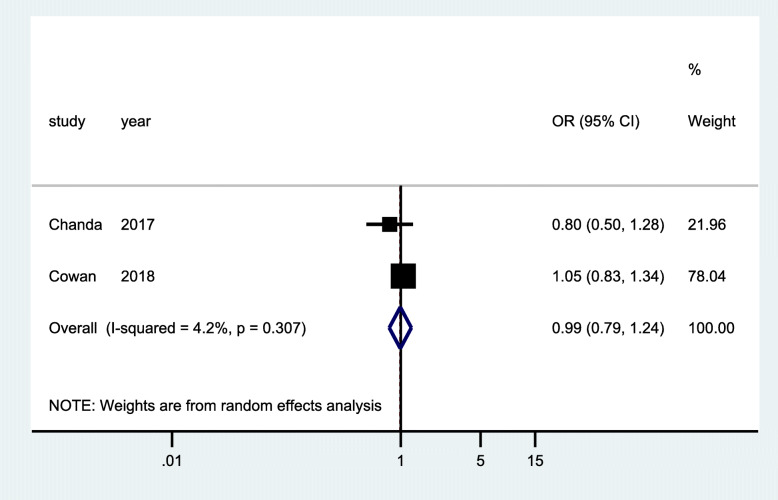
Forest plots of RCT studies with data on community-based interventions for HIV diagnosis

**Fig. 4:**
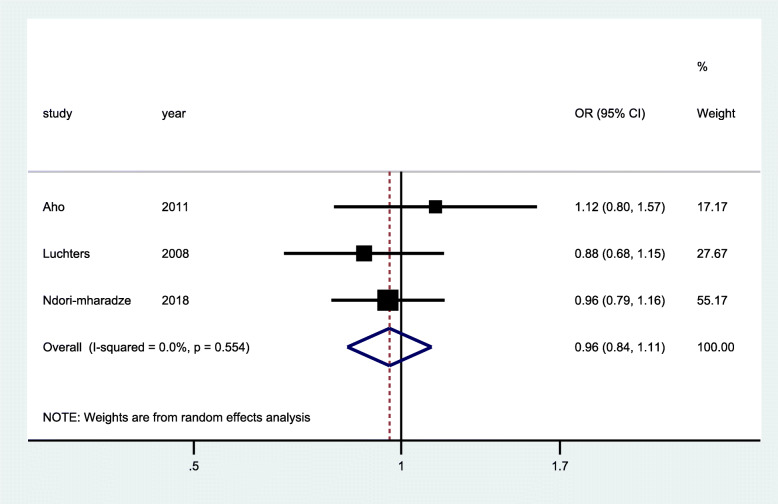
Forest plots of cross-sectional studies with data on community-based interventions for HIV diagnosis

HIV diagnosis was measured in six cross-sectional studies [28, 39–43], two randomized controlled trials [29, 32], and one quasi-experimental study [37]. The quasi-experimental study (Fig. 2) was conducted in three different countries (Burundi, Cote d’Ivoire, and DRC) and measured the proportions of people living with HIV during the implementation of an enhanced peer outreach approach (EPOA) for the three countries. Pooled analysis of data from the three countries showed statistically significant increase in proportion of participants diagnosed with HIV (OR 2.23; 95% CI 1.23–4.05; *p* <0.001). However, pooled analysis of data from two RCTs (Fig. 3) that randomized participants to standard of care testing versus general peer support augmented with additional community mobilization showed that there was no significant improvement in HIV diagnosis among FSWs (OR 0.99; 95% CI 0.79–1.24; *p* = 0.307). Similarly, data from the pooled analysis of three cross-sectional studies (Fig. 4) showed that there was a reduction in HIV diagnosis tending towards the negative impact, although this was not statistically significant (OR = 0.96; 95% CI 0.84–1.11; *p* = 0.554). The community-based interventions comprised of peer-mediated service delivery to improve access of HIV services, intensified community mobilization, and integrated adapted health services to suit specific needs of FSW.

### Linkage to care

**Fig. 5:**
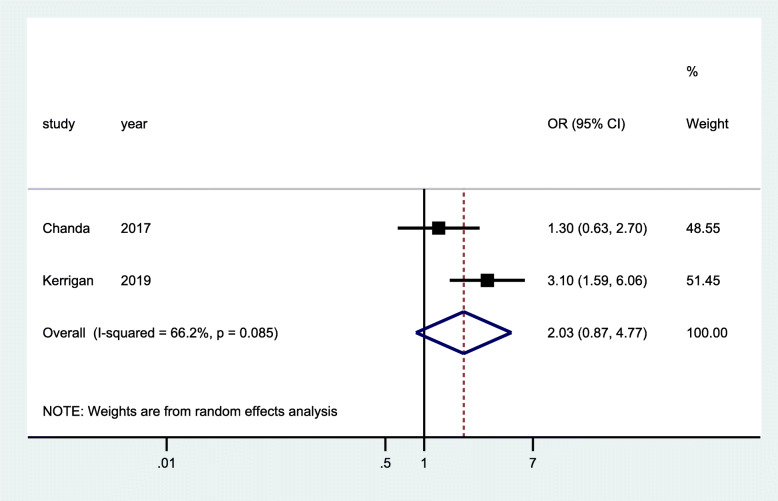
Forest plots of RCTs studies with data on community-based interventions for linkage to HIV care

**Fig. 6:**
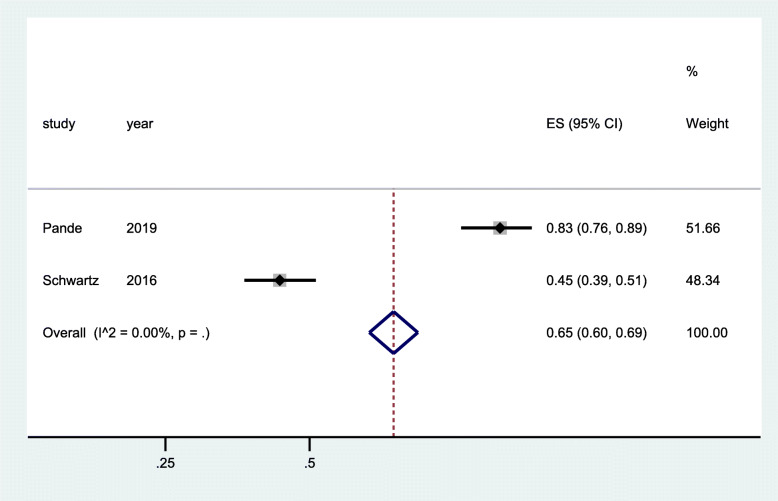
Forest plots of cross-sectional studies with data on community-based interventions for linkage to HIV care

Two RCTs [29, 35] and two cross-sectional studies [42, 43] reported linkage to care as an outcome. The data from pooled analysis of RCTs showed that there was improved linkage to care showing a tendency towards a positive impact of the intervention for the FSW who were provided with services in community-led drop-in centers, through venue-based peer education, introduction of social support, and text messages to promote solidarity and engagement. Nonetheless, this was not statistically significant (OR 2.03; 95% CI 0.87–4.77; *p* = 0.085) (Fig. 5)*.* The data of pooled analysis from the cross-sectional studies showed that there was a 65% improvement in linkage to care (overall estimate = 0.65; 95% CI 0.60–0.69; *p* = 0.000). This was observed at the end of the follow-up period of implementing health worker-led HIV service delivery in clinics based in hotspots and outreaches in community settings as well as peer-to-peer mechanism (Fig. 6)*.*

### Use of antiretroviral therapy and retention in care

**Fig. 7:**
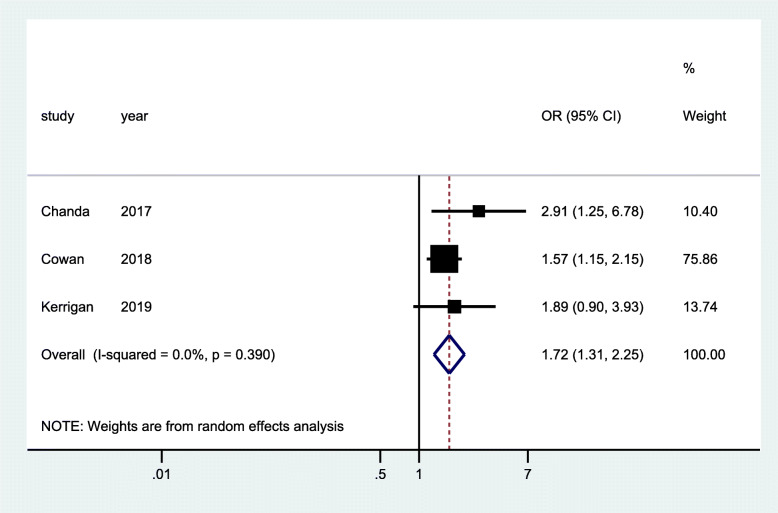
Forest plots of RCT studies with data on community-based interventions for ART use

**Fig. 8:**
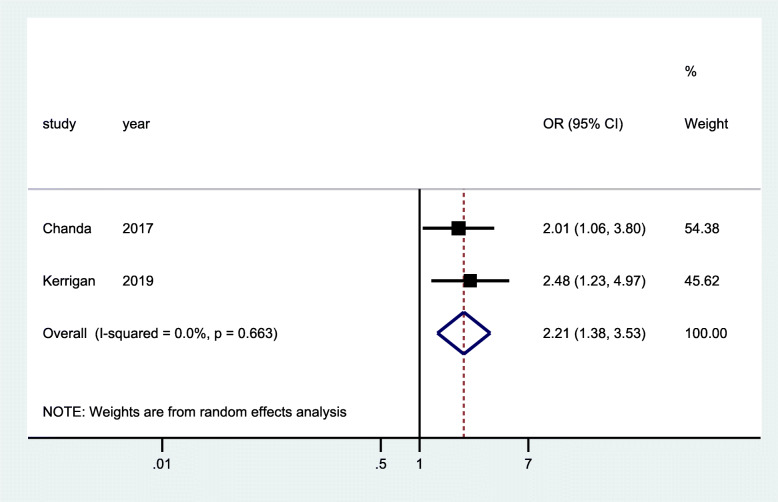
Forest plots of RCT studies at follow-up with data on community-based interventions for ART use

**Fig. 9:**
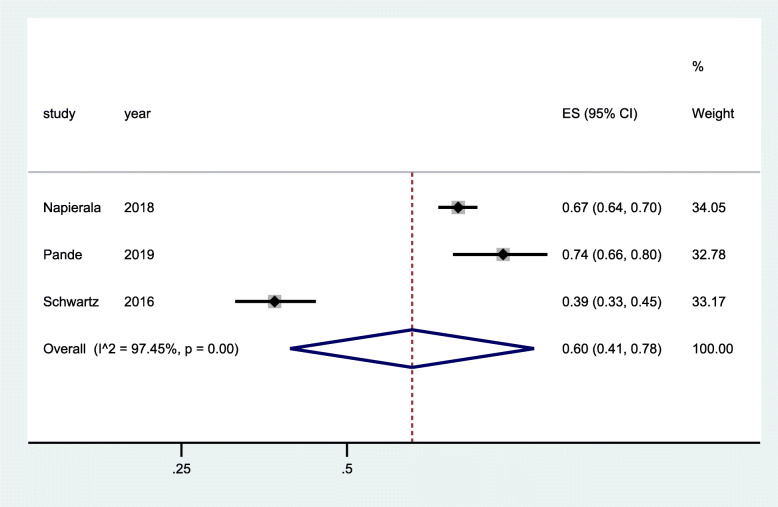
Forest plots of cross-sectional studies with data on community-based interventions for ART use

ART use was measured in three RCTs [29, 32, 35] and three cross-sectional studies [40, 42, 43]. One of the RCTs had a two-time point measurement at 1 month and 4 months of ART initiation [29] with 1 year of follow-up period, while the two RCTs [32, 35] had a 2-year follow up period with two-time point measurement at 0 months and 18–24 months. The data from pooled analysis of the three RCTs showed significant increase in odd of ART use (OR 1.72; 95% CI 1.31–2.25) at the start of the implementation of the intervention (Fig. 7) and a much higher increases in odds of ART use at the end of the follow-up period (OR 2.21; 95% CI 1.38–3.53). No heterogeneity was observed in both analyses (Figs. 7 and 8). The data from pooled analysis of three cross-sectional studies showed a 60% improvement of ART use with statistical significance (95% CI 0.41–0.78, *p* < 0.001) (Fig. 9). However, there was evidence of significant heterogeneity between studies (*I*^2 = 97.45%), as such we need to interpret these results with caution. The community intervention implemented for cross-sectional studies encompassed provision of targeted HIV services for FSW with augmented additional community mobilization activities and strengthened support networks.

### Viral suppression

Viral suppression was reported in three studies, two RCTs [32, 35] and one cross-sectional study [46]. All the three studies reported data during implementation, while one RCT also reported effect at the end of follow-up period [35]. The RCT by Cowan et al. [32] reported viral suppression rates for the participants that were assigned to clusters of usual care and those of the intervention clusters. At the end of the assessment period, 72% (588/828) in the intervention cluster showed minimal difference in reduction of viral load to undetectable level less than 1000 copies per mL, compared with 68% (590/869) of the participants in the usual care clusters. Similarly, in the RCT by Kerrigan et al. [35], the viral suppression rates showed slight improvement in the intervention group 40.0 (*n* = 36) to 50.6% (*n* = 46) compared to the control group 35.9 (*n* = 28) to 47.4% (*n* = 36) at the end of 18-month follow-up period. For the cross-sectional study [40], viral suppression was reported separately for young women aged 18–24 years and older women aged ≥25. Among the older FSW, 79% showed viral load suppression to undetectable level compared to the 62% of younger FSW, after both groups received targeted HIV services [46].

## Discussion

The objective of this systematic review was to gather evidence on the effectiveness of community-based interventions that provided HIV services to FSWs across the HIV care cascade in sub-Saharan Africa. We aimed to describe the community-based interventions that contributed to the proportions of FSW who tested for HIV, got diagnosed and linked to care, initiated on ART, and achieved viral suppression. Overall, this review found significant evidence on the impact of some community-based intervention packages on improved HIV testing, HIV diagnosis, and ART use. In the pooled analysis of all types of studies included in this review, we documented non-significant improvement in linkage to care and viral load suppression (Table 3). Many of the interventions were small-scale, research-based, and with limited time-bound implementation periods. A few non-research based and large-scale HIV prevention efforts had limited systematic means of monitoring outcomes along the HIV care cascade and therefore less methodical attention to constantly reviewing the effectiveness of interventions and altering delivery strategies accordingly.

**Table 3:** A summary of reported outcomes across the care and treatment cascade for included studies

(#Ref) Author (year)	HIV testing	HIV diagnosis	Linkage to care	ART use	Viral suppression
[28] Aho et al. (2012)	√	√			
[29] Chanda et al. (2017)		√	√	√	
[30] Cowan et al. (2019)	√	√		√	√
[32] Cowan et al. (2018)		√		√	√
[33] Huet et al. (2011)		√		√	√
[34] Kelvin et al. (2019)	√				√
[35] Kerrigan et al. (2019)	√				
[36] Lafort et al. (2018)			√	√	√
[36] Lafort et al. (2018)	√		√		
[37] Lillie et al. (2019)	√	√			
[38] Lafort et al. (2016)		√		√	
[39] Luchters et al. (2008)		√			
[40] Napierala et al. (2018)	√	√		√	√
[47] Ndori-mharadze et al. (2018)		√		√	
[42] Pande et al. (2019)	√	√	√	√	
[43] Schwartz et al. (2017)		√	√	√	
[44] Tun et al. (2019)			√	√	
[45] Vu et al. (2020)		√	√		

### HIV testing

The findings from this review on HIV testing show that community-based interventions which increased access to HIV testing are those that applied combined strategies such as incentivized peer network referrals, intensified FSW mobilization, structured follow-up to improve repeat testing, integrated health services adapted to specific needs of FSWs such as placing lubricants in clinics, and testing in static clinics based in hotspot [28, 29, 34]. The trend of improvement, however, was short-lived with proportions of testing reducing from 95 to 84% in just 4 months [29] and from 92 to 53% at 1 year follow-up [28]. According to these studies, the possible explanation for reduced proportions in testing over time was the saturation in target areas. However, the testing guidelines [2] indicate that FSWs should test every 3 months; therefore, the explanation of saturation may not be satisfactory. There is need to investigate why the effectiveness of such interventions may not be sustained to create opportunities for designing optimal interventions.

This review complements other studies [48–50] which showed that strategies may not work separately but rather are more effective when combined. In this review, all studies whose control groups did not have any enhanced strategies, did not realize improved HIV testing access [29, 34] although the rate of impact was the same across all interventions at the end of the follow-up period [29].

### HIV diagnosis

In regard to HIV diagnosis, the intervention that significantly identified more undiagnosed FSWs is the enhanced peer outreach model. This model involved training the FSWs that were naïve to peer network activities and used them to mobilize from their networks [37]. All the other studies in this review used the enhanced interventions similar to the ones used for HIV testing; however, there was no increase in HIV diagnosis [28, 29, 32, 39, 41].

The findings in this review are related to other studies conducted in Ukraine [51] and Malawi [52], though these were conducted in the general population. Both studies used the newly diagnosed patients to refer people in their networks for testing, and the HIV diagnosis results were promising. With this finding, we emphasize that interventions are unique and may impact cascade stages differently. Therefore, to maximize impact, service delivery models need to be routinely monitored and reviewed to ascertain their effectiveness and revise the strategies accordingly.

### Linkage to care

In this review, we have documented that the data from pooled analysis showed no significant improvement in linkage to care. However, we note that programs that had a positive effect on linkage are those that provided a combination package of HIV services delivered in areas with close proximity to high concentrations of FSWs such as hotspots and drop-in centers alongside extended working hours during evenings, weekends, and those that carried out sensitivity training for all the service providers [35, 42]. Previous reviews have indicated that, for optimal linkage to care, FSWs need to access comprehensive HIV services alongside structural interventions. Such structural interventions include those that focus to address stigma and discrimination, violence prevention, and legal challenges associated with practicing sex work [17, 43, 53, 54]. Notably, it was not clear from this review if such interventions were integrated in the service provision for FSWs in hotspots and drop-in centers.

These findings also indicate that irregular and mobile provision of HIV services poses challenges of follow-up for linkage in care. Health workers may not maintain constant engagement with FSWs, and therefore, there is need for strengthening the community systems that allow health workers and peers to constantly engage and provide HIV services in the community for FSW.

### Use of antiretroviral therapy

Similar to other outcomes, effective interventions for ART also provided a broad package of interventions, but the models in the specific packages varied for each outcome. For example, ART use was improved if interventions were provided in the static facility services based in hotspots, but the services had to be provided on a daily basis and on flex hours with room for phone consultation [42]. In addition, ART use was improved by interventions that had adherence support and use of text message follow-up as well as police sensitivity trainings to reduce violence [32, 35, 46]. However, increased ART use could not be sustained up to the end of the follow-up period [29, 32, 35]. Notably, we found only one country, Zimbabwe, that had national program with dedicated FSW clinics [46]. This finding implies that most of the interventions were on a small-scale and research-based with limited resources, and this could possibly explain why increased ART use was not sustained among FSWs.

Attaining short-term ART use by FSW has been reported in another systematic review [55], where it is indicated that 10% of FSW were stopping ART every year and cumulatively the proportions of ART interruption would increase. These findings imply that, for sustained ART use, a combination of behavioral-, biomedical-, and structural-related strategies should be uniquely tailored to the needs and priorities of FSWs and implemented as part of the established health systems with adequate financing.

### Viral suppression

In this review, all the programs whose primary outcome was to improve viral suppression implemented interventions that strengthened engagement in HIV care by FSWs; however, they did not lead to significant reduction in viral loads [32, 35, 40]. Although non-significant, the effect showed a positive trend of undetectable viral load suppression resulting from the exposure to the interventions. Failure to attain significant improvements in viral load suppression could have been due to gaps in community systems for supporting adherence to ART reported in some of the studies [32, 40]. The challenges of improving viral load suppression among FSWs have been reported in various research intervention studies. For example, studies conducted in sub-Saharan Africa reported that only 40 to 82% of FSW achieved viral suppression after exposure to various intervention programs [33, 56, 57].

One of the studies in this review compared the progression of viral load suppression between young FSWs aged 18–24 years and FSWs older than 25 years. Seventy-eight percent (78%) of the older FSWs attained higher rates of viral suppression, compared to 62% young FSWs. Young FSWs face unique underlying social and structural problems than older FSWs, such as more severe social and economic challenges, child protection issues, and mistrust and competition among older and young FSWs, a situation that may hinder access to services designed for adult FSWs [58–60].

These findings indicate that FSWs have varying challenges within their subgroups, and as such, interventions must consider the unique needs of different subgroups of FSWs. For example, programs must conduct intentional engagement of segments or subgroups of FSWs in designing and planning interventions. There should be optimal involvement of FSWs in activities such as mapping hotspots, identifying the FSW categories working in specific areas, assessing their risk and tailoring interventions according to the risks. This will enable implementation of interventions that are likely to be more effective along all the stages of HIV care cascade and lead to the ultimate goal of viral suppression and improved quality of life.

### Limitations of the study

In this review, only one quasi-experimental study was included. Inclusion of only one type of design and comparing it with RCTs and observational designs may bias the overall effect. Above all, quasi-experimental designs do not eliminate the problem of confounding variables. Indeed, while all other studies in this review showed no difference in the diagnosis outcome, we only observed a positive effect on the quasi study design. The results of diagnosis could be due to the fact that participants allocated to the different models of interventions were somewhat dissimilar. Further, in this review, we included studies whose primary aim was not to measure the effect of community intervention models, but rather we considered the reported results of our outcome of interest. For such studies, the effect may have been under or overestimated since critical observations on the processes of implementation were not emphasized. Finally, out of the 18 studies included in this review, only four were RCTs; we may therefore be underpowered to conclude on which models in community-based interventions were more effective than the others.

## Conclusion

Overall, the evidence brought forward from this review shows that the effects of community-based interventions vary across the different stages of HIV care cascade. A broad package of interventions including a combination of behavioral, biomedical, and structural, designed with specific strategies, unique to each cascade stage appear to be more effective. Data suggests that there are limited community-based interventions that increase HIV diagnosis; however, a number of interventions are effective for HIV testing and ART use and can also increase linkage to care and viral suppression although not significantly. This review also found that the positive effects of community interventions to increasing cascade outcomes are short term, and the implementation is mostly done in research settings. As such, the information on long-term treatment outcomes and the extent to which FSWs link to, adhere on ART and get virally suppressed is sparse.

This review observed challenges related to expanding the community-based interventions for FSWs, as majority of interventions were small scale. Considerations for governments to strengthen support and integrate community-based HIV services in the mainstream health system are paramount. As countries roll out differentiated service delivery models, the health systems need to focus on provision of sustainable equitable care for all subgroups of FSWs. This can however be effective if program data on FSW care cascade is actively collected and reported to inform programming for HIV services targeting FSWs. Further, there is need to conduct sufficiently powered research on the effectiveness of community-based interventions on the HIV care cascade for FSWs, to identify evidence-based optimal interventions and components and guide resource allocation.

## Data Availability

All data generated or analyzed during this study are included in this manuscript.

## Abbreviations

AIDS: Acquired immune deficiency syndrome
ART: Antiretroviral therapy
CINAHL: Cumulative Index of Nursing and Allied Health Literature
EMBASE: Excerpta Medica database
FSW: Female sex workers
GRADE: Grading of Recommendations Assessment Development and Evaluation
HIV: Human immunodeficiency virus
IAS: International AIDS Society
ICASA: International Conference on HIV/AIDS and Sexually Transmitted Infections in Africa
KP: Key populations
MEDLINE: Medical Literature Analysis and Retrieval System Online
MOH: Ministry of Health
NOS: New Castle Ottawa scale
PRISMA-P: Preferred Reporting Items for Systematic Reviews and Meta-Analysis Protocols
PROSPERO: Prospective Register of Systematic Reviews
PubMed: Public/Publisher MEDLINE
ROBINS-I: Risk of Bias in Non-Randomized Studies of Interventions
SSA: Sub-Saharan Africa
STIs: Sexual transmitted infections
UAC: Uganda AIDS Commission
UNAIDS: United Nations Programme on HIV/AIDS
WHO: World Health Organization
